# A Comprehensive School-Based Mental Health Model: A Decade in the Making

**DOI:** 10.3390/bs15101428

**Published:** 2025-10-21

**Authors:** Wendy M. Reinke, Keith C. Herman, Aaron Thompson, Sarah Owens

**Affiliations:** 1 Department of Educational, School & Counseling Psychology, University of Missouri, Columbia, MO 65211, USA; hermanke@missouri.edu (K.C.H.); owenssar@missouri.edu (S.O.); 2 Missouri Prevention Science Institute, University of Missouri, Columbia, MO 65211, USA; thompsonaa@health.missouri.edu

**Keywords:** school mental health, school partners, systems level change

## Abstract

Over the past decade, researchers in partnership with school practitioners developed a comprehensive school mental health model. The model includes a universal screening system that incorporates teacher and student reports on areas of risk known to be linked to mental health issues in youth. The Early Identification System (EIS) was developed as a feasible and socially valid universal screener that allows schools to use data to identify universal prevention interventions, areas for professional development for staff, and students who would benefit from selective or indicated interventions. The EIS can also be used to monitor change over time. Originally developed as part of a Coalition of six school districts, the US Department of Education invested in this comprehensive school mental health model to be adapted for rural schools. This article describes the partnership between school practitioners, the use of the model over time, and research conducted over the past decade. Implications for practice and policy are discussed.

## 1. A Comprehensive School-Based Mental Health Model: A Decade in the Making

According to the Center for Disease Control (CDC), youth in the US are experiencing a mental health crisis (Centers for Disease Control and Prevention, 2024). Since 2011, there has been an increasing trend in youth experiencing symptoms of depression. The most recent data from 2023 indicates that 40% of high school youth reported experiencing persistent feelings of sadness or hopelessness, a 10% increase since 2013 (Centers for Disease Control and Prevention, 2024). In addition, the percentage of high school students reporting that they seriously considered attempting suicide, made a suicide plan, and attempted suicide have all increased (Centers for Disease Control and Prevention, 2023). According to the World Health Organization (World Health Organization, 2021), one in seven children ages 10–19 years old experiences a mental health disorder with evidence that these problems are increasing. Mental health challenges in children and adolescents are real and common (World Health Organization, 2021), but also preventable and treatable. However, many youth who would benefit from services remain unidentified and do not receive support (Merikangas et al., 2010; Twenge et al., 2019).

Youth spend the greatest portion of their day in the school environment, making schools an optimal setting for both conducting evidence-based assessments and providing interventions to identify, prevent and intervene in youth mental health problems (Herman et al., 2019, 2021b; Kilgus et al., 2015; Reinke et al., 2018, 2021). One planful way for schools to increase their ability to provide mental health supports is by using a public health model of service delivery. A public health model offers services across three tiers that increase in intensity at each level: (1) universal, (2) selective, and (3) indicated support for the delivery of evidence-based practices and interventions (EBIs; see Merrell & Buchanan, 2006; Stormont et al., 2012). Universal supports are designed to proactively and preventatively address the needs of all students, with the goal of meeting the needs of approximately 80% of the student population. These supports also help reduce the severity of challenges experienced by students at higher risk. Selective interventions are provided to a smaller group—typically 5–10% of students—who require additional support beyond what is offered universally. For the 1–5% of students whose needs persist despite receiving universal and selective interventions, more intensive and individualized indicated interventions are implemented. The 80-15-5 ratio is a generalization that helps illustrate the public health pyramid, a tiered framework for organizing and prioritizing prevention efforts, and provides a way to allocate resources effectively across different levels of risk. This tiered approach is commonly known as Multi-Tiered System of Supports (MTSS; McIntosh & Goodman, 2016) in educational settings. The flexibility of this model allows for effective use of school resources and seamless supports for students.

One important component of a comprehensive school mental health model that uses tiered intervention supports is a universal social emotional behavior (SEB) screening system. Universal SEB screening, classified under universal supports within the MTSS framework, helps to identify students who may benefit from more intensive mental health supports (Reinke et al., 2023). Additionally, universal screening data can be used to inform universal prevention and professional development topics for school staff by identifying strengths and areas of need within a school setting (see Reinke et al., 2018). Further, when conducted regularly, universal screening data can be used to monitor progress in areas of need at the school or grade levels over time.

Using universal SEB screening data within the context of a comprehensive tiered model of prevention and intervention supports can produce positive outcomes for students. Yet, despite the availability of many technically adequate SEB screening instruments, schools rarely use them (Brann et al., 2022). In fact, recent surveys of district and school administrators suggest that the number of schools using universal SEB screening may be as low as between 5–10% (Briesch et al., 2022; Dineen et al., 2022). Potential reasons for the low use of universal SEB screening in schools include a host of barriers that are associated with the usability of the measures (Herman et al., 2023). These usability barriers include the financial costs of screeners; lack of time, resources, and infrastructure to administer and interpret them; excessive number of students identified in need of services; and lack of clear connection of screening results to guide interventions and supports (Herman & Bonifay, 2023).

Thus, if schools are to be instrumental in reducing the burden and prevalence of youth mental health issues, we need to work toward creating systematic, feasible, and sustainable models of school mental health. The purpose of this manuscript is to describe a comprehensive school mental health model that was developed in partnership between school personnel and researchers to overcome common challenges and barriers to the use of universal SEB screening within a multi-tiered framework. This comprehensive model to identify, prevent, and intervene in youth mental health issues is fully described, including the progression of the model over time and initial evidence to support the impact of this model on youth mental health.

## 2. Formation of the School Mental Health Partnership

In 2012, mental health experts and community members from our local county were well aware of the emerging youth mental health crisis. County citizens, influenced by the canvassing of community mental health activists, passed a ¼ cent county-wide sales tax to fund mental health services for children. As a result, schools across the six school districts within the county were flooded with offers from private mental health service providers to embed their services within schools. While well intentioned, these efforts would likely have led to disjointed and uncoordinated services. The six independent school districts in the county were aware of the potential of the new funding stream to improve outcomes for students, and the superintendents met to discuss methods of maximizing the benefits to youth in the county. They reached out to researchers from the university with expertise in school mental health, prevention science, and implementation science.

**School Mental Health Coalition.** A Coalition was formed between university partners, the six independent school districts, and local parochial schools. To begin with, the Coalition conducted a needs assessment by surveying school personnel. The needs assessment revealed widespread SEB concerns across all grade levels. It also identified the absence of a systematic process for identifying students in need of support and a lack of professional development focused on addressing student mental health needs. In response to these findings and through collaborative discussions, the Coalition developed and submitted a proposal for funding through the local youth mental health tax. The goal was to strengthen the infrastructure of schools and to establish a comprehensive school mental health model.

**Development of the Early Identification System.** The first step to building a comprehensive school mental health model was to identify or develop a universal SEB screener that would overcome the common barriers in conducting and using screening data in schools. The Coalition considered using an already existing measure but had concerns. In particular, the cost of available screeners was prohibitive, many costing as much as $6 per student. In addition, many of the nationally normed screeners take extensive time for teachers to complete, some do not have a comparable student report available, and they are prone to over-identifying the number of students at risk, overwhelming the resources of schools (see Herman et al., 2023). As such, the Coalition decided to develop their own measure. In collaboration with university scientific partners, the Coalition chose to develop the Early Identification System (EIS), a universal SEB screening measure that has both a teacher and student report, that was locally normed to avoid over-identification, and that would be free to the 55 schools across the 6 school districts. Locally normed measures utilize the data within each school building to determine which students have elevated risk compared to their peers. These data are standardized among the student data for the school, with students indicated as having elevated risk when they are two standard deviations above the mean level of risk for their peers—this allows schools to identify the students most in need of supports within their building rather than comparing this risk to a national sample. Further, the screener needed to be comprehensive in nature, in that it identified malleable risk factors that we know if intervened upon early can prevent long-term less retractable mental health issues. As such these malleable risk factors were also linked to evidence-based interventions (EBIs) that were known to reduce the identified risk if implemented with fidelity.

Risk indicators to produce risk domains and items for the EIS were identified from the extant literature and related to negative SEB, and academic outcomes. The developmental cascades theory informed the selection of risk domains and related items (Burt et al., 2021; Masten et al., 2005; Patterson et al., 1989). This theory suggests that early social behavior challenges can lead to additional, co-occurring difficulties over time. Indicators related to inattention, academic competence, peer relationships, internalizing and externalizing problems, emotion dysregulation, and school disengagement were included because of their frequent co-occurrence with social difficulties and risk for academic failure. Additionally, experiences of being bullied and engaging in bullying were incorporated, as both are high-risk factors strongly linked to severe negative outcomes, including suicide attempts and death (Gini & Espelage, 2014; Li et al., 1997).

The selected risk indicators are all well-established, modifiable factors linked to poor academic and social behavioral outcomes. For example, externalizing behaviors, peer rejection, and deficits in social skills during childhood have been associated with adverse developmental trajectories in adolescence and early adulthood (LoParo et al., 2023; Mrug et al., 2012). Internalizing problems—among the most common emotional and behavioral challenges faced by youth—are also key contributors to negative outcomes (Racine et al., 2021). Inattention is another prevalent concern among school-aged children, with strong associations to both academic underachievement and social-emotional difficulties (Zentall, 2005; Herman & Ostrander, 2007; Herman et al., 2008). These SEB risk factors often co-occur with academic struggles, and experiences of academic failure can, in turn, exacerbate behavioral and emotional challenges. As such, identifying early signs of risk is crucial to prevent the emergence or escalation of these problems. Notably, students with academic risk are more likely to exhibit concurrent behavioral and social concerns (Darney et al., 2013; Reinke et al., 2008). In sum, the identified risk factors are consistently linked with serious academic, emotional, behavioral, and social challenges.

**Figure 1:**
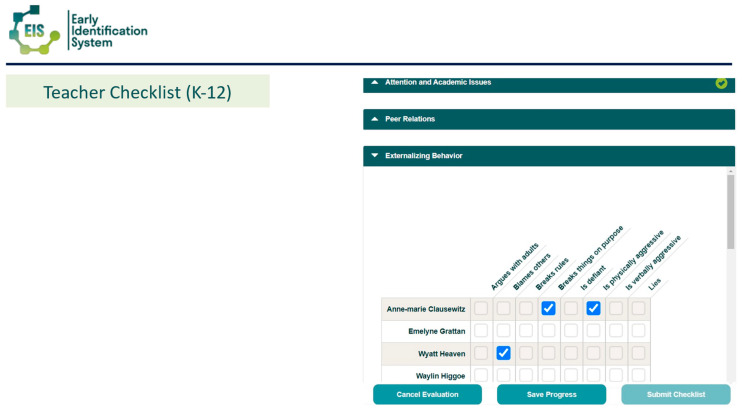
Sample EIS-TR Teacher Checklist. From the Early Identification System, by W.M. Reinke, K.C. Herman, and A.M. Thompson. Copyright [2015] by W.M. Reinke, K.C. Herman, A.M. Thompson. Reprinted with permission.

**Figure 2:**
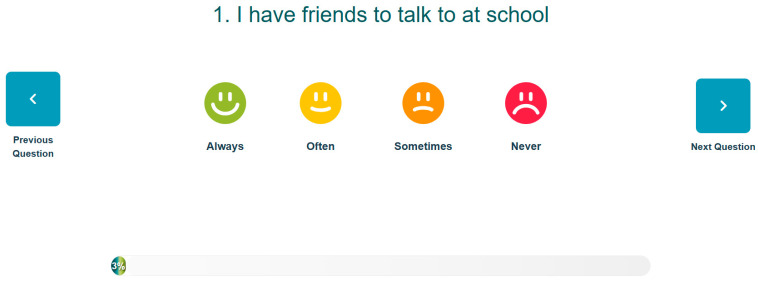
Sample EIS—SR Student Item. From the Early Identification System, by W.M. Reinke, K.C. Herman, and A.M. Thompson. Copyright [2015] by W.M. Reinke, K.C. Herman, A.M. Thompson. Reprinted with permission.

The EIS has both a teacher-report and student self-report. To overcome the burden of the time required by teachers to complete universal screening, the EIS teacher report (EIS-TR), asks teachers to check a box for each risk indicator only if a student is exhibiting the risk. The checklist has a list of risk indicators by domain with a list of student names down the side (see Figure 1 for sample of the teacher checklist). This is different from other screeners, which often ask teachers to complete a survey of many items on a scale from never to always, one student at a time (typically taking hours to complete for a classroom of students). The EIS-TR only takes teachers in elementary classroom about 10 min and secondary teachers slightly longer depending on how many students they are asked to rate. Starting in 3rd grade (3rd-12), students are asked to complete a survey of 37 items on a scale of *never, sometimes, often, or always*, taking them between 5–15 min (see Figure 2 for sample of the student survey). Both the teacher and student report are done electronically using the EIS during a brief window (often 2 weeks). When the cycle closes and all assessments are complete, the system produces reports that can be used by the school-based mental health providers and problem-solving teams.

**Use of the Data.** The EIS is administered two to three times per year across grades K–12, with student self-report data collected from grades 3 through 12. Data are gathered electronically via a secure server and linked to an interactive dashboard. Because all participating schools utilize the same data system, results can be aggregated and analyzed at various levels—school, district, and county. For example, county-level data can be reviewed to identify broader trends and emerging areas of concern among youth. At the same time, each school has access to its own dashboard, allowing users to examine data at the school-wide, grade-level, and individual student levels.

Results are presented using a public health framework: risk levels are color-coded—red indicates high risk, yellow signals some risk, and green reflects areas of strength. Specifically, when 20% or more of students meet criteria for a particular risk indicator, the dashboard displays red, suggesting the need for universal intervention. A yellow flag appears when 15-19% of students are identified, indicating elevated but not critical levels of concern. Green signifies that fewer than 15% of students show risk in a particular domain, highlighting it as a relative strength (e.g., strong peer relationships). Reviewing grade-level data often helps schools identify where universal interventions may be especially warranted—whether at the grade or classroom level.

**Figure 3:**
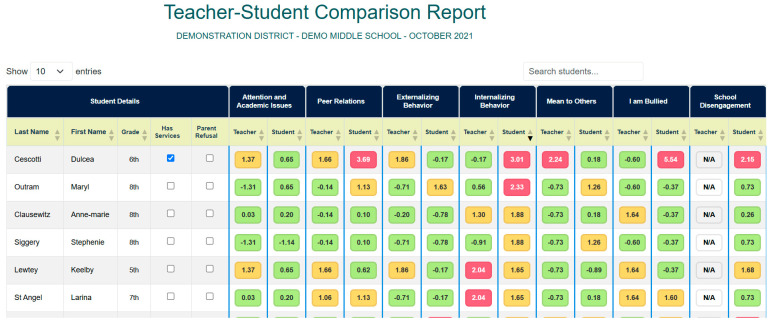
Sample Teacher-Student Comparison Report. From the Early Identification System, by W.M. Reinke, K.C. Herman, and A.M. Thompson. Copyright [2015] by W.M. Reinke, K.C. Herman, A.M. Thompson. Reprinted with permission.

**Figure 4:**
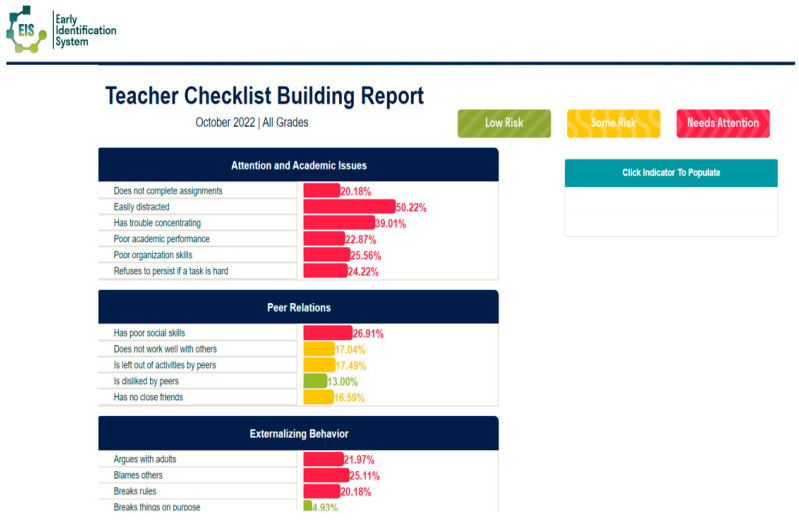
Sample Teacher School Level Report Used to Identify Areas for Universal Prevention. From the Early Identification System, by W.M. Reinke, K.C. Herman, and A.M. Thompson. Copyright [2015] by W.M. Reinke, K.C. Herman, A.M. Thompson. Reprinted with permission.

Following data collection, schools using the EIS will review reports to guide both individual intervention and school level supports. Schools begin by reviewing the individual risk for internalizing problems and being bullied. Problem solving teams can easily look at the teacher-student comparison report (see Figure 3) to sort students from highest to lowest based on risk for internalizing problems and being bullied. The teacher-school comparison reports and individual student report show which students have elevated risk based on whether they are two standard deviations or higher on risk in a domain compared to peers in their school (red), one standard deviation above (yellow) or within the normal range (green). Schools then follow-up with these students accordingly, checking in for risk of suicidal ideation and whether bullying interventions need to commence. Schools also look at school-wide reports to determine areas of need for universal prevention efforts and possible areas for professional development for staff (see Figure 4 for an example of a teacher school level report). For instance, if the teacher school report indicates more than 20% of students in the building have poor organizational skills, a universal intervention to support students in organizing materials for completing and turning in assignments may be warranted.

**Figure 5:**
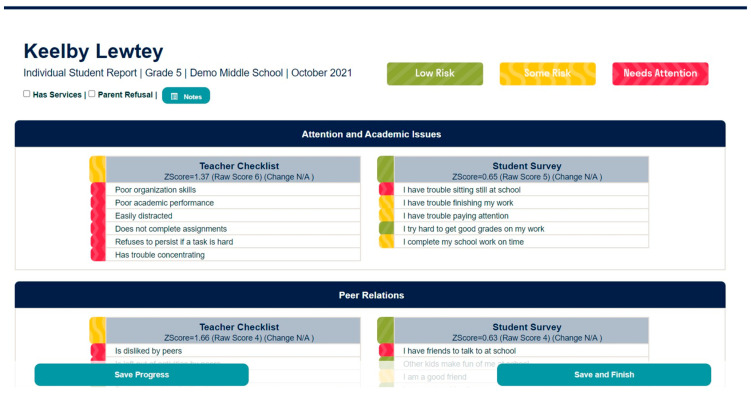
Sample Individual Student Report. From the Early Identification System, by W.M. Reinke, K.C. Herman, and A.M. Thompson. Copyright [2015] by W.M. Reinke, K.C. Herman, A.M. Thompson. Reprinted with permission.

Schools can also review individual student reports, which summarize the data from both the teacher and student in a single report (see Figure 5 for a sample individual student report). Schools can readily access individual student reports either by typing in the name of the student or through links from other reports. For instance, school problem solving teams can sort internalizing problems for students with highest risk to lowest in the teacher-student comparison report. Then, they can click on the name of a student listed on this report, and it takes them directly to the individual student report where they can see more specifically how items on the EIS were reported both by the teacher and by the student to identify areas of strength and concern.

**Validation of the Early Identification System (EIS).** Funding from the US Department of Education, Institute of Education Sciences (R305H170023) in the form of an education partnership grant was obtained and used to validate the EIS and conduct an initial evaluation of the social validity and impact of the comprehensive school mental health model.

Multiple studies have demonstrated that the EIS-SR possesses strong psychometric properties across elementary (Reinke et al., 2022), middle (Thompson et al., 2021), and high school (Herman et al., 2021a) populations. Across these studies, seven subscales consistently showed adequate factor loadings, supporting the measure’s structural validity. Reinke et al. (2022) also provided evidence of concurrent validity, with the internalizing problems, attention and academic issues, emotion dysregulation, and school disengagement subscales correlating significantly with comparable subscales from the Behavior Assessment System for Children–Third Edition (BASC-3; Kamphaus & Reynolds, 2015). Moreover, fall EIS-SR subscale scores were predictive of key spring outcomes, including attendance, disciplinary referrals, bullying victimization, and academic achievement in math and reading. More recently, the EIS-TR has also been shown to be reliable and valid across grade levels (Reinke et al., under review). Importantly, both the EIS-SR and EIS-TR have demonstrated measurement invariance across grade level, gender, race, and socioeconomic status, indicating that the measures are psychometrically sound and function equivalently across diverse student groups.

A recent study examined both the usability and social consequences of implementing the EIS, addressing a critical concern given that schools often cite poor usability as a primary barrier to adopting universal screening practices. Herman et al. (2023) evaluated EIS implementation across 54 K–12 schools, involving a sample of 23,104 students. The study found that nearly all schools, teachers, and students successfully completed the EIS as planned. Schools utilized the resulting data to provide a range of support—including universal, selective, and indicated interventions—to a substantial proportion of students identified as at risk. Additionally, the screening data informed professional development planning for educators. Notably, 79% of schools implemented the comprehensive mental health model with high fidelity (i.e., gathering and using universal screening data, identifying and implementing EBIs, monitoring progress). Implementation fidelity was not associated with student demographic characteristics, indicating that schools serving predominantly minoritized student populations were just as likely to implement the EIS effectively. These findings suggest that the EIS may address many of the common usability challenges that have historically limited the widespread adoption of behavioral screening tools in schools.

**EIS Single Item Predictors.** In addition to the seven risk domains, the EIS also includes several one-item indicators that have been shown to be very powerful in predicting later negative outcomes for youth. For instance, the EIS-TR includes two items that ask teachers to indicate if a student is not academically or not behaviorally ready for their grade. These items were adapted from the Kindergarten Academic and Behavior Readiness Scale (K-ABR; Stormont et al., 2017). The K-ABR uses single items of teacher report in the fall of kindergarten of student readiness, either behaviorally or academically. The measure has been shown to predict spring academic and behavior outcomes, as well as spring first grade outcomes for students (see Stormont et al., 2017, 2019). More recently a longitudinal investigation found that using the EIS, teachers can identify students as early as the fall of kindergarten who are at risk for social behavioral challenges and lower reading and math scores in 3rd grade (Reinke et al., 2025b). In this study, students who were not behaviorally ready in kindergarten had twice the odds of having elevated risk for teacher and student report of externalizing problems, teacher reported attention and academic issues, and student reported school disengagement, as well as more office discipline referrals in 3rd grade than peers who were behaviorally ready. Students who were not academically ready had two times the odds of having elevated risk for teacher report of attention problems in 3rd grade. These items allow schools to quickly identify students who are not ready for their grade and target them for early support to potentially prevent later social and academic problems.

Another single item that was added to the EIS-SR by requests from our partnering school counselors was to ask students “do you have an adult you can talk to at school if you need help”. A recent study investigated whether students who reported not having a trusted adult to talk to at school had elevated risk for internalizing problems. Self-report from students in elementary, middle, and high school (N = 13,881) indicated that not having a trusted adult to talk to at school was strongly associated with elevated internalizing symptoms across the school year and was associated with worsening internalizing symptoms over time for middle and high school students (Reinke et al., 2025a). Many of our schools use these data to identify students without a trusted adult and provide targeted interventions to ensure students feel supported and have someone to talk to at school by the end of the year. One of our rural schools recently reported that they used these data from the fall to identify students without a trusted adult and then through problem solving teams identified adults who could connect to these youth. The adults then actively worked to build rapport with each student. They reported by the spring administration of the EIS that no students reported not having a trusted adult.

**Figure 6:**
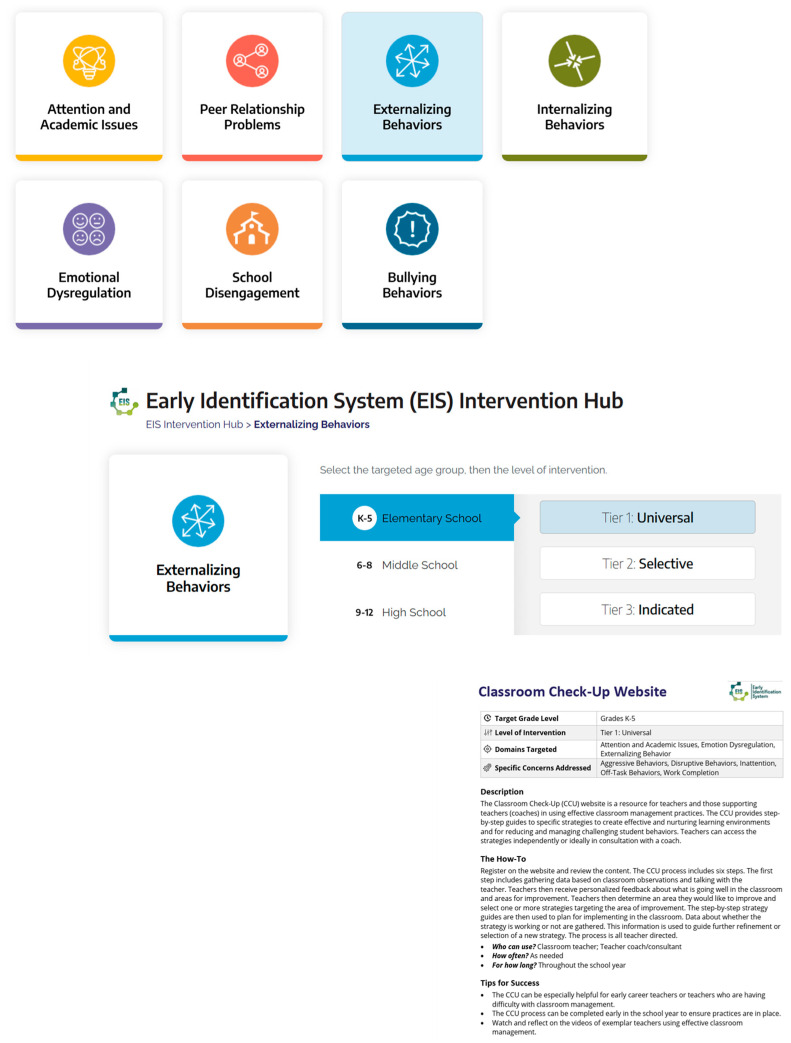
Example From the EIS Intervention Hub. Copyright [2019] by W.M. Reinke, K.C. Herman, A.M. Thompson. Reprinted with permission.

**Aligning EIS Data with EBIs.** One additional area that makes the EIS relevant and important to schools is the ability to connect the EIS screening data to EBIs that we know will reduce risk in the identified domain if implemented with fidelity. In the early years of the Coalition work, the team began developing a menu of options of EBIs that could be linked to the screening data. This menu of options was developed as a word document that described the EBI and linked the team to resources. More recently, with funding from the US Department of Education, Institute of Education Sciences (R305C190014) we were able to build what is referred to as the EIS Intervention hub. This is a resource developed as part of the National Center for Rural School Mental Health. The National Center for Rural School Mental Health was funded by the Institute of Education Sciences as a research and development center to support the expansion and adaptation of the work we were doing with the Coalition for rural schools across the US. Over the past seven years, the National Center for Rural School Mental Health has been working with partners from rural schools in Missouri, Montana, Virginia and several other states to scale up the comprehensive school mental health model using the EIS and building implementation supports that can be used by any school, particularly those with low resources. As a result, the IES Intervention hub, which is free for use by any school (see https://ruralsmh.com/intervention-hub, accessed on 15 October 2025) lists EBIs that are categories by the EIS data risk domains, for elementary, middle, and high school, and by tier (universal, selective, or indicated). School problem solving teams or mental health professionals can visit the EIS Intervention Hub and select the area in need of support. A menu of EBI options appears, and schools can peruse one-page descriptions of each intervention or practice to determine fit with the school context. These one-page descriptions also link schools directly to resources to implement the EBIs they choose (see Figure 6 for visual of how the EIS Intervention Hub links to EBIs). Schools can search the site to find free resources, practices, and manualized interventions.

## 3. Initial Evaluations of the Comprehensive School Mental Health Model

Through our funding with the US Department of Education, Institute of Education Sciences (R305H170023) we conducted a study to investigate the longitudinal outcomes of the comprehensive school mental health model among our Coalition schools. In this study, data from the EIS-SR were used to examine the trajectories of student-reported mental health concerns over a three-year period (Reinke et al., 2022). Contrary to national trends reported by the Centers for Disease Control and Prevention (2023), which indicate increasing mental health challenges among youth, this study found an overall decline in social, emotional, and behavioral problems during the study period. Using Growth Mixture Modeling, four distinct student trajectories emerged: (1) students with consistently high levels of problems, (2) students with decreasing problems, (3) students with increasing problems, and (4) students with stable, low levels of problems. Importantly, these trajectories were linked to implementation fidelity; students in schools with lower fidelity were more likely to fall into the increasing problems group. Although the study did not utilize a randomized controlled design, the results provide encouraging evidence for the effectiveness of the EIS model, particularly when implemented with high fidelity.

## 4. Scaling of the Comprehensive School Mental Health Model

**Figure 7:**
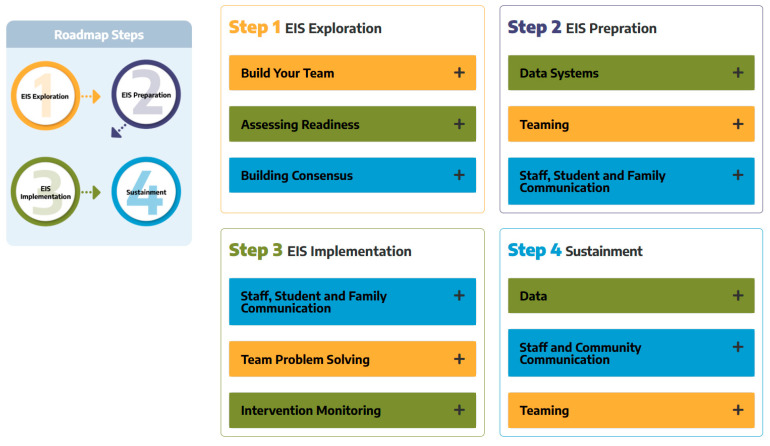
Visual of the Implementation Roadmap. Copyright [2019] by W.M. Reinke, K.C. Herman, A.M. Thompson. Reprinted with permission.

The National Center for Rural School Mental Health has been working to support the scaling of the comprehensive school mental health model with an emphasis on supporting rural schools. The Center provides national leadership and outreach to support the dissemination of the model. Among these dissemination efforts, the Center has developed a cost calculator to support schools in learning about the cost/benefit of school-based programs. The Center has also developed the EIS Intervention Hub, mentioned earlier, which has a menu of evidence-based programs and practices across seven areas of risk, allowing schools to link data from the EIS screener to evidence-based practices that if done with fidelity are known to reduce or prevent the identified area of risk. Lastly, the Center has developed a professional development model to support rural school implementation of the EIS model, including an Implementation Roadmap (see Figure 7 for visual of the Implementation Roadmap stepped process), and an Implementation Hub which includes free assessment tools for progress monitoring, monitoring fidelity, and tracking pre-post outcomes of intervention supports provided to students. These resources are intended to help schools adopt, implement, evaluate, and sustain the comprehensive school mental health model. All of these implementation resources are currently free to any school (see ruralsmh.com).

The Center is currently in the last year of a randomized control trial evaluating the impact of the comprehensive school mental health model on student outcomes. The 2026 school year will mark the last year of the trial. At the time of this paper 79 rural schools have participated in the trial with an anticipated 35 more in the final year. Each school was randomized to receive the intervention which included the EIS and monthly consultation. Those schools randomized to the waitlist control condition, completed the EIS without receiving supports until the end of the school year. Control schools received monthly consultation the following school year. Schools who participated in the trial had the option to continue using the EIS after completion at no cost. Most schools have chosen to continue, finding the EIS highly feasible, relevant, and useful to their schools. We hope to continue to scale this comprehensive school mental health model nationally. We anticipate that findings from our current randomized trial will be positive, especially given the ongoing and positive work that we have conducted over the past 10 years in our Coalition schools.

## 5. Implications for Practice and Policy

If schools are to effectively reduce the population prevalence of youth mental health concerns, we need comprehensive school mental health models that overcome challenges and barriers schools face in implementation. Over the past decade, we have developed, implemented, and adapted our model to fit the needs of schools. Each year, in response to our school partners, we improve our systems and support based on feedback. More recently, we have incorporated student voices into our model, such that we have a high school advisory board and a team of youth researchers who work alongside our team. School mental health researchers cannot stay abreast of school issues or innovate without partnering with school personnel and youth. Over the past decade we have seen changes to the school landscape, social context, and presenting problems. By partnering, our model is ever improving to mitigate ongoing and new challenges. Also, our partners have helped our EIS to be efficient and effective. For instance, we regularly solicit input on how to improve the system from school partners. This has resulted in the EIS we have today. To be ready for scale, the EIS must demonstrate that it can be implemented with fidelity across diverse school contexts, sustain equitable reach, and produce reliable results for all student groups. It must also show that schools can feasibly act on screening data with clear protocols for linking results to support, while maintaining affordability and long-term sustainability (see Gottfredson et al., 2015). The National Center for Rural School Mental Health recently held a conference where school partners provided feedback and youth researchers presented focus group data they had gathered from other youth. From this information we are continuing to improve our EIS and develop professional development materials in collaboration with our youth researchers for adults in school buildings. These are valuable opportunities that need to be incorporated into this work. More importantly, the work on the comprehensive school mental health model that has been done over the past decade, and which has impacted thousands of youth and school personnel, could not have occurred without the support of local and federal funding. Investing in improving youth outcomes is investing in the future of our country.

## 6. Conclusions

Over the past decade, partnerships between schools, researchers, and youth have created a feasible yet comprehensive school mental health model. This work is needed now more than ever to meet the rising mental health risks of youth in the US. Unfortunately, too few schools currently have access to usable universal SEB screening and support systems. Our goal over the next decade is to change these circumstances by making the EIS accessible to every school in the country that wants to use it.

## Data Availability

No new data were created or analyzed in this study.

## References

[B1-behavsci-15-01428] Brann K. L., Daniels B., Chafouleas S. M., DiOrio C. A. (2022). Usability of social, emotional, and behavioral assessments in schools: A systematic review from 2009 to 2019. School Psychology Review.

[B2-behavsci-15-01428] Briesch A. M., Chafouleas S. M., Dineen J. N., McCoach D. B., Donaldson A. (2022). School building administrator reports of screening practices across academic, behavioral, and health domains. Journal of Positive Behavior Interventions.

[B3-behavsci-15-01428] Burt S. A., Clark D. A., Gershoff E. T., Klump K. L., Hyde L. W. (2021). Twin differences in harsh parenting predict youth’s antisocial behavior. Psychological Science.

[B4-behavsci-15-01428] Centers for Disease Control and Prevention (2023). Youth risk behavior survey data summary and trends report: 2011–2021.

[B5-behavsci-15-01428] Centers for Disease Control and Prevention (2024). Youth risk behavior survey data.

[B6-behavsci-15-01428] Darney D., Reinke W. M., Herman K. C., Stormont M., Ialongo N. (2013). Children with co-occurring academic and behavior problems in 1st grade: Distal outcomes in 12th grade. Journal of School Psychology.

[B7-behavsci-15-01428] Dineen J. N., Chafouleas S. M., Briesch A. M., McCoach D. B., Newton S. D., Cintron D. W. (2022). Exploring social, emotional, and behavioral screening approaches in US public school districts. American Educational Research Journal.

[B8-behavsci-15-01428] Gini G., Espelage D. L. (2014). Peer victimization, cyberbullying, and suicide risk in children and adolescents. JAMA Pediatrics.

[B9-behavsci-15-01428] Gottfredson D. C., Cook T. D., Gardner F. E., Gorman-Smith D., Howe G. W., Sandler I. N., Zafft K. M. (2015). Standards of evidence for efficacy, effectiveness, and scale-up research in prevention science: Next generation. Prevention Science.

[B10-behavsci-15-01428] Herman K. C., Bonifay W. (2023). Best practices for examining and reporting the social consequences of educational measures. School Psychology.

[B11-behavsci-15-01428] Herman K. C., Lambert S. F., Reinke W. M., Ialongo N. S. (2008). Low academic competence in first grade as a risk factor for depressive cognitions and symptoms in middle school. Journal of Counseling Psychology.

[B12-behavsci-15-01428] Herman K. C., Ostrander R. (2007). The effects of attention problems on depression: Developmental, academic, and cognitive pathways. School Psychology Quarterly.

[B13-behavsci-15-01428] Herman K. C., Reinke W. M., Huang F. L., Thompson A. M., Doyle-Baker L. (2021a). Investigating the psychometric properties of the Early Identification System—Student Report in a middle school sample. School Psychology.

[B14-behavsci-15-01428] Herman K. C., Reinke W. M., Thompson A., Hawley K. (2019). The Missouri Prevention Center: A multidisciplinary approach to reducing the societal prevalence and burden of youth mental health problems. American Psychologist.

[B15-behavsci-15-01428] Herman K. C., Reinke W. M., Thompson A., Huang F., Owens S. (2023). Usability and social consequences of the Early Identification System as a universal screener for social, emotional, and behavioral risks. School Psychology.

[B16-behavsci-15-01428] Herman K. C., Reinke W. M., Thompson A. M., Hawley K. M., Stormont M. (2021b). Reducing the societal prevalence and burden of youth mental health problems: Lessons learned and next steps. School Psychology Review.

[B17-behavsci-15-01428] Kamphaus R. W., Reynolds C. R. (2015). Behavior assessment system for children—Third edition (BASC-3).

[B18-behavsci-15-01428] Kilgus S. P., Reinke W. M., Jimerson S. R. (2015). Understanding mental health intervention and assessment within a multi-tiered framework: Contemporary science, practice, and policy. School Psychology Quarterly.

[B19-behavsci-15-01428] Li G., Ling J., Discala C., Nordenholtz K., Sterling S., Baker S. P. (1997). Characteristics and outcomes of self inflicted pediatric injuries: The role of method of suicide attempt. Injury Prevention.

[B20-behavsci-15-01428] LoParo D., Fonseca A. C., Matos A. P., Craighead W. E. (2023). A developmental cascade analysis of peer rejection, depression, anxiety, and externalizing problems from childhood through young adulthood. Research on Child and Adolescent Psychopathology.

[B21-behavsci-15-01428] Masten A. S., Roisman G. I., Long J. D., Burt K. B., Obradović J., Riley J. R., Boelcke-Stennes K., Tellegen A. (2005). Developmental cascades: Linking academic achievement and externalizing and internalizing symptoms over 20 years. Developmental Psychology.

[B22-behavsci-15-01428] McIntosh K., Goodman S. (2016). Integrated multi-tiered systems of support blending RTI and PBIS.

[B23-behavsci-15-01428] Merikangas K. R., He J. P., Burstein M., Swanson S. A., Avenevoli S., Cui L., Benjet C., Georgiades K., Swendsen J. (2010). Lifetime prevalence of mental disorders in US adolescents: Results from the National Comorbidity Survey Replication–Adolescent supplement (NCS-A). Journal of the American Academy of Child & Adolescent Psychiatry.

[B24-behavsci-15-01428] Merrell K. W., Buchanan R. (2006). Intervention Selection in School-Based Practice: Using Public Health Models to Enhance Systems Capacity of Schools. School Psychology Review.

[B25-behavsci-15-01428] Mrug S., Molina B. S., Hoza B., Gerdes A. C., Hinshaw S. P., Hechtman L., Arnold L. E. (2012). Peer rejection and friendships in children with attention-deficit/hyperactivity disorder: Contributions to long-term outcomes. Journal of Abnormal Child Psychology.

[B26-behavsci-15-01428] Patterson G. R., DeBaryshe B. D., Ramsey E. (1989). A developmental perspective on antisocial behavior. American Psychologist.

[B27-behavsci-15-01428] Racine N., McArthur B. A., Cooke J. E., Eirich R., Zhu J., Madigan S. (2021). Global Prevalence of depressive and anxiety symptoms in children and adolescents during COVID-19: A meta-analysis. JAMA Pediatrics.

[B28-behavsci-15-01428] Reinke W. M., Herman K. C., Huang F., Glenn-Perez A., Raut P., Aguayo D., Venkat S., Boddie D., Harris J., Owens S. (2025a). Having a trusted adult at school: Concurrent and predictive relations with internalizing problems across development. Journal of Positive Behavior Interventions.

[B29-behavsci-15-01428] Reinke W. M., Herman K. C., Huang F., McCall C., Holmes S., Thompson A., Owens S. (2022). Examining the factor structure and concurrent and predictive validity of the early identification system—Student report in an elementary school sample. Journal of School Psychology.

[B30-behavsci-15-01428] Reinke W. M., Herman K. C., Petras H., Ialongo N. (2008). Empirically-derived subtypes of child academic and behavior problems: Co-occurrence and distal outcomes. Journal of Abnormal Child Psychology.

[B31-behavsci-15-01428] Reinke W. M., Herman K. C., Thompson A., Evans S. W., Owens J. S., Bradshaw C. P., Weist M. D. (2023). Scaling-up screening of students’ behavioral and mental health needs. Handbook of school mental health—Innovations in science and practice.

[B32-behavsci-15-01428] Reinke W. M., Herman K. C., Thompson A., McCall C., Copeland C., Holmes S., Owens S. (2021). Investigating the longitudinal association between fidelity to a large-scale comprehensive school mental health prevention and intervention model and student outcomes. School Psychology Review.

[B33-behavsci-15-01428] Reinke W. M., Herman K. C., Thompson A., Shaoli S. S., Bhuiyan P. (under review). Evaluating a pragmatic universal social behavioral screener: Confirmatory factor analysis, invariance testing, and predictive validity of the Early Identification System—Teacher report.

[B35-behavsci-15-01428] Reinke W. M., Stormont M., Herman K. C. (2025b). Teacher reported readiness in kindergarten predicting 3rd grade outcomes. Advances in Mental Health.

[B34-behavsci-15-01428] Reinke W. M., Thompson A., Herman K. C., Holmes S., Owens S., Cohen D., Tanner-Jones L., Henry L., Green A., Copeland C., County Schools Mental Health Coalition (2018). The county schools mental health coalition: A model for community level impact. School Mental Health.

[B36-behavsci-15-01428] Stormont M., Cohen D. R., Herman K. C., Reinke W. M. (2019). Teacher-rated school readiness items in a Kindergarten sample: Outcomes in first grade. School Psychology.

[B37-behavsci-15-01428] Stormont M., Reinke W. M., Herman K. C., Lemke E. (2012). Academic and behavior supports for at-risk students: Tier 2 interventions.

[B38-behavsci-15-01428] Stormont M., Thompson A., Herman K. C., Reinke W. M. (2017). The social and emotional dimensions of a single item overall school readiness screener and its relations with academic outcomes. Assessment for Effective Intervention.

[B39-behavsci-15-01428] Thompson A., Huang F., Smith T., Reinke W. M., Herman K. C. (2021). Confirmatory factor structure and predictive validity of the early identification system-student report in a community sample of high school students. School Mental Health.

[B40-behavsci-15-01428] Twenge J. M., Cooper A. B., Joiner T. E., Duffy M. E., Binau S. G. (2019). Age, period, and cohort trends in mood disorder indicators and suicide-related outcomes in a nationally representative dataset, 2005–2017. Journal of Abnormal Psychology.

[B41-behavsci-15-01428] World Health Organization (2021). Adolescent mental health.

[B42-behavsci-15-01428] Zentall S. (2005). Theory and evidence-based strategies for children with attentional problems. Psychology in the Schools.

